# Effects of Sodium-Glucose Cotransporter 2 Inhibitors on Metabolic Parameters in Patients With Type 2 Diabetes: A Chart-Based Analysis

**DOI:** 10.14740/jocmr2467w

**Published:** 2016-01-26

**Authors:** Hisayuki Katsuyama, Hidetaka Hamasaki, Hiroki Adachi, Sumie Moriyama, Akiko Kawaguchi, Akahito Sako, Shuichi Mishima, Hidekatsu Yanai

**Affiliations:** a Department of Internal Medicine, National Center for Global Health and Medicine Kohnodai Hospital, Chiba, Japan

**Keywords:** Body weight, HbA1c, Liver function, Sodium-glucose cotransporter 2 inhibitor

## Abstract

**Background:**

Effects of the new class of anti-diabetic drugs, sodium-glucose cotransporter 2 (SGLT2) inhibitors, on metabolic parameters in patients with type 2 diabetes remain largely unknown.

**Methods:**

We retrospectively picked up patients who had been continuously prescribed SGLT2 inhibitors for 1 month or more between April 2014 and November 2015 by a chart-based analysis, and compared the data before the SGLT2 inhibitor treatment with the data at 1, 2, 3 and 6 months after the SGLLT2 inhibitor treatment started.

**Results:**

Fifty patients were eligible for the analyses in our study. The HbA1c levels as well as body weight significantly decreased at 1, 2, 3 and 6 months after the start of SGLT2 inhibitors. Systolic blood pressure tended to decrease only at 1 and 2 months, but there was no change at 3 and 6 months. No significant change was observed in serum high-density lipoprotein-cholesterol (HDL-C), triglyceride (TG), low-density lipoprotein-cholesterol (LDL-C) and non-HDL-C levels. Serum aspartate aminotransferase (AST) and alanine aminotransferase (ALT) levels significantly decreased at 3 and 6 months after the prescription. The hematocrit levels significantly increased at 1, 2, 3 and 6 months, and the estimated glomerular filtration rate (eGFR) levels significantly decreased at 1 month after the start of SGLT2 inhibitors. A significant correlation between reductions in HbA1c levels and HbA1c levels at baseline was observed at 1, 3 and 6 months. The decreases in serum ALT levels were also significantly correlated with the baseline ALT levels at 3 and 6 months.

**Conclusion:**

Present study demonstrated that SGLT2 inhibitors significantly reduced HbA1c and body weight and improved liver functions, whereas no significant change was observed in serum lipid profiles.

## Introduction

Sodium-glucose cotransporter 2 (SGLT2) is expressed in the proximal tubule of kidney and mediates reabsorption of approximately 90% of the filtered glucose load [1]. The SGLT2 inhibitors block reabsorption of filtered glucose by inhibiting SGLT2, and promote the renal excretion of glucose without requiring insulin secretion or action [2]. Various clinical trials showed that SGLT2 inhibitors improved the glycemic control as monotherapy and combination therapy with anti-diabetic medicines including insulin with a low risk of hypoglycemia [3-5]. It was also reported that SGLT2 inhibitors have various favorable effects on cardiovascular (CV) risk factors including reduction of body weight and blood pressure [6]. Moreover, a recent investigation has demonstrated that empagliflozin reduced CV events defined as a composite of death from CV causes, non-fatal myocardial infarction, or non-fatal stroke [7]. These insights may indicate that the SGLT inhibitors exert multifactorial beneficial effects on CV risks and reduce CV events.

Since April 2014, ipragliflozin, dapagliflozin, luseogliflozin, canagliflozin, tofogliflozin and empagliflozin have been approved and widely used to date in Japan. However, the reports evaluating the effects of SGLT2 inhibitors in clinical practice are limited. Here, we retrospectively studied the effects of SGLT2 inhibitors on metabolic parameters in patients with type 2 diabetes.

## Materials and Methods

### Subjects

This study was approval by the Institutional Ethics Committee in National Center for Global Health and Medicine (NCGM-G-001910-00), and was also performed in accordance with the Declaration of Helsinki.

We selected patients with type 2 diabetes, who had been prescribed SGLT2 inhibitors for 1 month or longer between April 2014 and November 2015 based on medical charts. We compared the data at baseline and at 1, 2, 3 and 6 months after the start of SGLT2 inhibitors. Body weight, blood pressure, plasma glucose, HbA1c, serum low-density lipoprotein-cholesterol (LDL-C), triglyceride (TG), high-density lipoprotein-cholesterol (HDL-C), non-HDL-C, aspartate aminotransferase (AST) and alanine aminotransferase (ALT) in studied subjects were measured almost at the same time point at the baseline and at 1, 2, 3 or 6 months after the start of SGLT2 inhibitors. LDL-C levels were determined by the direct measurement. Estimated glomerular filtration rate (eGFR) was calculated by a modified three variable equation for estimating GFR in Japanese patients [8]. Since SGLT2 inhibitors have similar chemical structures, we analyzed the subjects taking all kinds of SGLT2 inhibitors.

Comparison of the variables determined before and after was analyzed by a paired Student’s *t*-test. Pearson’s simple correlations coefficients were performed to determine the correlations between the data before the start of SGLT2 inhibitor treatment and changes in variables after the SGLT2 inhibitor treatment. All data are expressed as mean ± SD. P < 0.05 was considered to be statistically significant.

## Results

We found 72 patients who had taken SGLT2 inhibitors at least once between April 2014 and November 2015. Nineteen patients were excluded due to lack of the data after the start of SGLT2 inhibitors, and three patients were also excluded since they had already taken SGLT2 inhibitors at baseline. Thus, 50 patients were analyzed in this study.

Table 1 shows the baseline characteristics of the studied subjects. The mean body mass index (BMI) was 32.1 kg/m^2^.

**Table 1:** Baseline Characteristics of the Study Subjects Who Were Prescribed SGLT2 Inhibitors (n = 50)

Age	48 ± 10
Sex (male/female)	18/32
Body height (cm)	161 ± 8
Body weight (kg)	84.9 ± 17.6
Body mass index (BMI) (kg/m^2^)	32.1 ± 5.9
Systolic blood pressure (mm Hg)	130 ± 15
Diastolic blood pressure (mm Hg)	80 ± 11
Plasma glucose (mg/dL)	180 ± 75
Hemoglobin A1c (%)	8.9 ± 2.0

Table 2 shows the SGLT2 inhibitors used in the studied subjects. Dapagliflozin was the most prescribed SGLT2 inhibitor, followed by luseogliflozin and ipragliflozin. Hypoglycemic or anti-hypertensive, or lipid lowering agents which subjects used during the studied period are shown in Table 3.

**Table 2:** SGLT2 Inhibitors Prescribed to the Subjects Studied at Baseline (n = 50)

Dapagliflozin 5 mg	16 (32%)
Ipragliflozin 25 mg	4 (8%)
Ipragliflozin 50 mg	10 (20%)
Tofogliflozin 20 mg	7 (14%)
Luseogliflozin 2.5 mg	11 (22%)
Canagliflozin 100 mg	1 (2%)
Empagliflozin 10 mg	1 (2%)

**Table 3:** Hypoglycemic, Anti-Hypertensive and Lipid Lowering Agents Which the Subjects Had Taken Before and After the Treatment With SGLT-2 Inhibitors

Medications	Pre-treatment (n = 50)	1 month (n = 50)	2 months (n = 41)	3 months (n = 37)	6 months (n = 23)
Biguanides	41	39	35	30	19
Thiazolidinediones	17	17	16	14	8
DPP-4 inhibitors	26	24	21	18	10
Sulfonylurea	11	9	6	5	4
α-GI	6	7	7	6	2
Glinides	2	1	1	1	2
GLP-1 analogues	7	8	7	6	9
Insulin	15	14	12	11	5
ACEi/ARB	19	20	18	16	11
Statins	30	30	27	22	13

ACEi: angiotensin converting enzyme inhibitor; ARB: angiotensin receptor blockers; α-GI: alpha-glucosidase inhibitor; DPP-4: dipeptidyl peptidase-4; GLP-1: glucagon-like peptide 1.

The changes of clinical parameters after the start of SGLT2 inhibitors are shown in Tables 4-7. The body weight significantly decreased by 1.7 kg at 1 month (P < 0.001), 1.9 kg (P < 0.001), 2.4 kg (P = 0.002) and 2.0 kg (P = 0.023) at 2, 3 and 6 months, respectively. The HbA1c levels also decreased by 0.8% (P < 0.001), 0.9% (P < 0.001), 1.0% (P < 0.001) and 1.3% (P = 0.001) at 1, 2, 3 and 6 months, respectively, after the start of SGLT2 inhibitors. Systolic blood pressure tended to decrease only at 1 and 2 months, but there was no change at 3 and 6 months. No significant change was observed in serum HDL-C, TG, LDL-C and non-HDL-C levels. Serum AST and ALT levels significantly decreased at 3 and 6 months. The hematocrit levels significantly increased at 1, 2, 3 and 6 months, and the eGFR levels significantly decreased at 1 month after the start of SGLT2 inhibitors.

**Table 4:** Changes in the Examined Variables After 1 Month of SGLT2 Inhibitor Administration (n = 50)

	n	Pre-treatment	1 month	P
Body weight (kg)	41	85.0 (18.7)	83.3 (18.1)	< 0.001
Systolic blood pressure (mm Hg)	31	129 (14)	124 (15)	0.126
Diastolic blood pressure (mm Hg)	31	80 (10)	73 (17)	0.014
Plasma glucose (mg/dL)	46	180 (78)	168 (81)	0.368
HbA1c (%)	41	9.0 (2.0)	8.2 (1.7)	< 0.001
HDL-C (mg/dL)	45	48 (11)	48 (12)	0.696
TG (mg/dL)	45	221 (153)	269 (291)	0.072
LDL-C (mg/dL)	21	120 (39)	121 (36)	0.905
Non-HDL-C (mg/dL)	37	142 (30)	149 (36)	0.110
AST (IU/L)	44	39 (27)	39 (31)	0.900
ALT (IU/L)	44	50 (40)	53 (48)	0.241
eGFR (mL/min/1.73 m^2^)	45	94 (36)	89 (31)	0.012
Hematocrit (%)	44	41.2 (4.2)	42.8 (4.4)	< 0.001

**Table 5:** Changes in the Examined Variables After 2 Months of SGLT2 Inhibitor Administration (n = 41)

	n	Pre-treatment	2 months	P
Body weight (kg)	28	86.2 (19.3)	84.3 (19.2)	< 0.001
Systolic blood pressure (mm Hg)	23	131 (13)	124 (15)	0.051
Diastolic blood pressure (mm Hg)	23	79 (10)	76 (10)	0.615
Plasma glucose (mg/dL)	34	187 (78)	161 (63)	0.006
HbA1c (%)	28	8.7 (1.6)	7.8 (1.4)	< 0.001
HDL-C (mg/dL)	33	47 (10)	47 (10)	0.713
TG (mg/dL)	33	197 (106)	189 (92)	0.567
LDL-C (mg/dL)	13	123 (46)	122 (41)	0.809
Non-HDL-C (mg/dL)	28	137 (28)	136 (32)	0.875
AST (IU/L)	33	42 (30)	35 (20)	0.051
ALT (IU/L)	32	54 (46)	49 (39)	0.149
eGFR (mL/min/1.73 m^2^)	33	95 (39)	92 (35)	0.052
Hematocrit (%)	31	41.0 (4.2)	42.8 (3.9)	0.003

**Table 6:** Changes in the Examined Variables After 3 Months of SGLT2 Inhibitor administration (n = 37)

	n	Pre-treatment	3 months	P
Body weight (kg)	31	84.4 (18.4)	82.0 (17.9)	0.002
Systolic blood pressure (mm Hg)	26	131 (15)	128 (16)	0.402
Diastolic blood pressure (mm Hg)	26	76 (10)	75 (9)	0.615
Plasma glucose (mg/dL)	35	189 (84)	160 (58)	0.032
HbA1c (%)	30	8.9 (2.0)	7.9 (1.6)	< 0.001
HDL-C (mg/dL)	32	49 (11)	51 (15)	0.195
TG (mg/dL)	32	199 (112)	197 (145)	0.922
LDL-C (mg/dL)	17	122 (40)	119 (37)	0.641
Non-HDL-C (mg/dL)	24	143 (29)	137 (30)	0.180
AST (IU/L)	32	40 (29)	31 (23)	< 0.001
ALT (IU/L)	32	50 (43)	43 (38)	0.033
eGFR (mL/min/1.73 m^2^)	33	98 (38)	94 (33)	0.137
Hematocrit (%)	32	41.1 (4.0)	43.1 (3.8)	< 0.001

**Table 7:** Changes in the Examined Variables After 6 Months of SGLT2 Inhibitor Administration (n = 23)

	n	Pre-treatment	6 months	P
Body weight (kg)	19	89.6 (19.1)	87.6 (19.1)	0.023
Systolic blood pressure (mm Hg)	15	132 (14)	131 (16)	0.896
Diastolic blood pressure (mm Hg)	15	78 (12)	80 (10)	0.465
Plasma glucose (mg/dL)	23	192 (97)	158 (62)	0.017
HbA1c (%)	20	8.8 (2.3)	7.5 (1.7)	0.001
HDL-C (mg/dL)	22	49 (12)	51 (16)	0.499
TG (mg/dL)	22	197 (108)	202 (110)	0.815
LDL-C (mg/dL)	10	118 (45)	102 (37)	0.162
Non-HDL-C (mg/dL)	16	142 (24)	140 (30)	0.791
AST (IU/L)	21	38 (21)	29 (13)	0.006.
ALT (IU/L)	21	46 (34)	38 (25)	0.035
eGFR (mL/min/1.73 m^2^)	23	97 (40)	94 (30)	0.511
Hematocrit (%)	22	41.9 (3.9)	43.8 (4.1)	< 0.001

**Figure 1:**
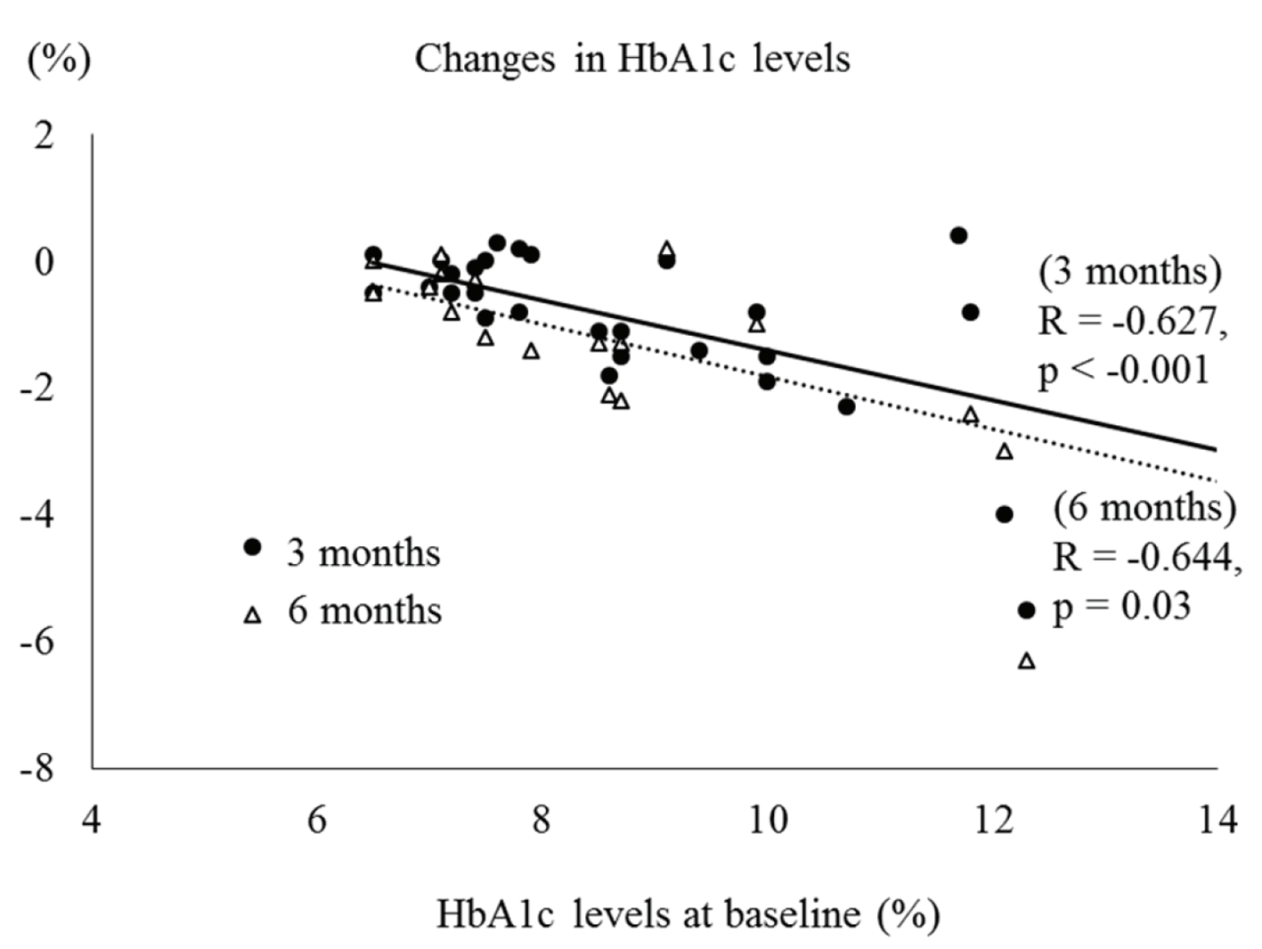
Correlation between HbA1c levels at baseline and changes in HbA1c levels.

**Figure 2:**
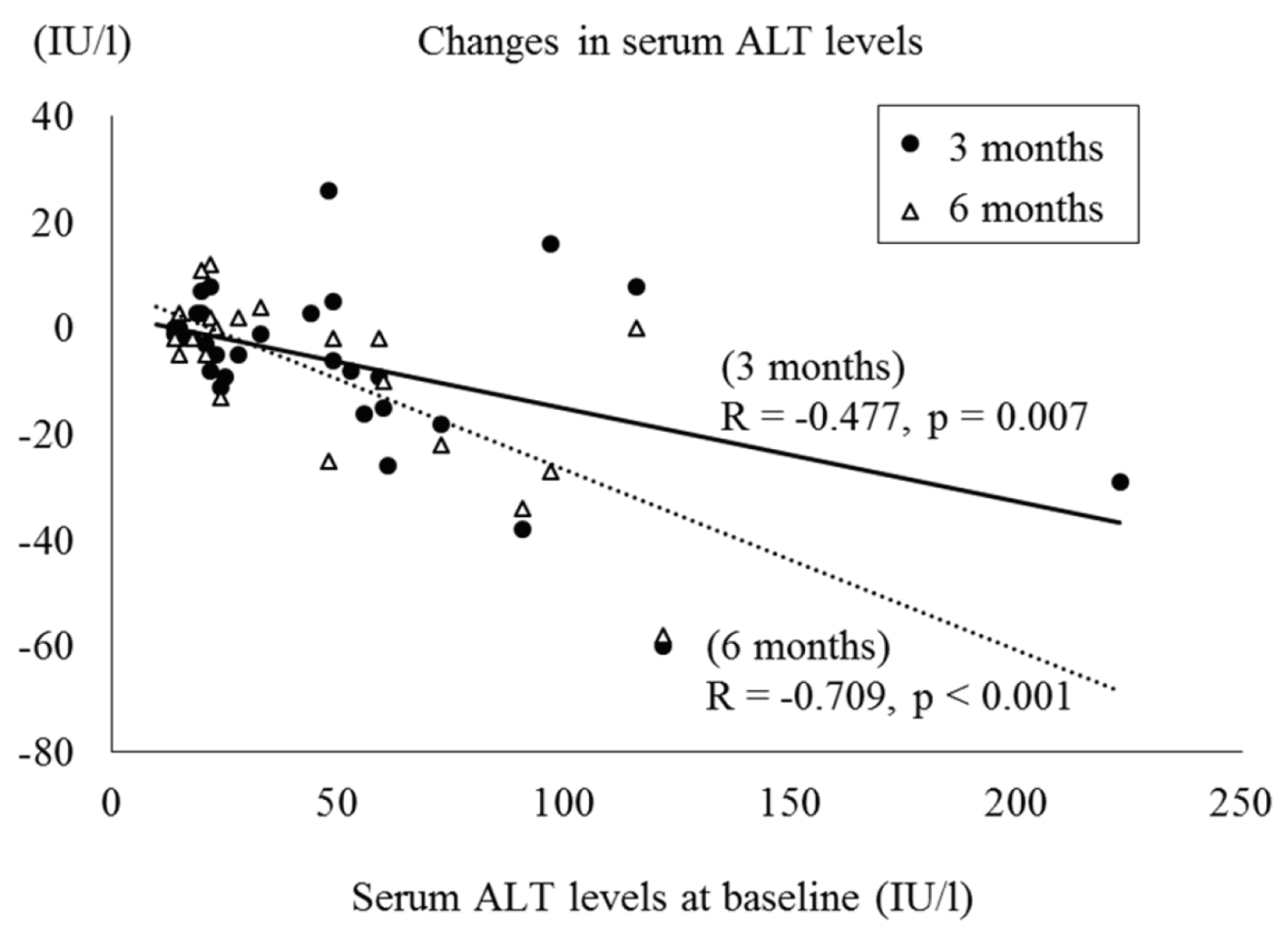
Correlation between serum ALT levels at baseline and changes in serum ALT levels.

We found a significant correlation between reductions in HbA1c levels and HbA1c levels at baseline and at 1, 3 and 6 months, respectively (R = -0.521, P < 0.001; R = -0.538, P = 0.006; R= -0.627, P < 0.001; R = -0.644, P = 0.003) (Fig. 1). Moreover, the decreases in serum ALT levels were negatively correlated with the baseline ALT levels at 3 and 6 months, respectively (R = -0.477, P = 0.007; R = -0.709, P < 0.001) (Fig. 2). On the other hand, no significant correlation was observed between the change in body weight and the baseline BMI.

## Discussion

In this study, we examined the efficacy of the SGLT2 inhibitors on metabolic parameters in patients with type 2 diabetes. The results revealed that SGLT2 inhibitors improved the glycemic control and reduced body weight as well as serum AST and ALT levels, whereas no significant changes were observed in serum lipid profiles.

Previous studies reported that SGLT2 inhibitors reduced HbA1c levels by approximately 0.5-0.7% compared to placebo [3, 9]. In our study, HbA1c levels decreased by 0.8-1.3%, which was greater than those in previous reports [3, 9]. As we demonstrated, HbA1c reduction by SGLT2 inhibitors was greater in patients with higher HbA1c levels at baseline. The baseline HbA1c in our study was 8.8%, which was higher as compared with previous studies [3, 9], which may explain the greater reduction in HbA1c levels.

In our study, the decrease of body weight was observed at 1 month and was sustained over 6 months. The results consist with previous reports. Clar et al reported that body weight reduction was sustained over 50 months in a meta-analysis [3]. Kaku et al also showed that dapagliflozin reduced body weight for 52 months by both monotherapy and add-on therapy [4]. Although body weight reduction by DPP-4 inhibitor, sitagliptin, was correlated with the baseline BMI in our previous study [10], such a correlation was not observed in this study. The reduction of body weight by SGLT2 inhibitors was reported to be also induced by osmotic diuretics [11], which may make a difference in the association of the baseline BMI to body weight reduction between DPP-4 inhibitors and SGLT2 inhibitors.

Previous reports also showed that osmotic diuretics by SGLT2 inhibitors lowered systolic blood pressure, and reduction of systolic blood pressure was sustained for 50 weeks compared to placebo [4, 11]. In our study, a tendency toward a decrease in systolic blood pressure was observed only at 1 and 2 months, and there was no significant change at 3 and 6 months. Although it is uncertain why sustained effects of SGLT2 inhibitors on systolic blood pressure were not observed in our study, the limited availability of data and lifestyle might have an influence on our results.

In spite of a significant reduction of body weight, no significant changes in serum lipids were observed in our study. Only a few studies reported the effects of SGLT2 inhibitors on serum lipid profile. Matthaei et al showed that 52-week treatment of dapagliflozin significantly increased serum HDL-C levels with no significant change of serum LDL-C and TG levels [12]. Monami et al also reported that SGLT-2 inhibitors significantly increased HDL-C levels with no change of TG and LDL-C levels [13]. The small increase in both LDL-C and HDL-C was observed in EMPA-REG OUTCOME trial [7]. Further studies will be needed to elucidate the effects of SGLT2 inhibitors on serum lipid metabolism.

In the present study, serum AST and ALT levels significantly decreased at 3 and 6 months. Moreover, the reductions in ALT levels showed a significant correlation with the baseline ALT levels at 3 and 6 months. To our knowledge, the effects of SGLT2 inhibitors on liver function in humans have not been reported yet. Tahara et al showed in high-fat diet- and streptozotocin-nicotinamide-induced type 2 diabetic mice, 4-week administration of ipragliflozin improved hepatic steatosis and reduced serum levels of aminotransferases [14]. Qiang et al reported that the treatment of luseogliflozin reduced the liver weight with lipid accumulation and serum ALT levels in non-alcoholic steatohepatitis model mice [15]. These results in animal model experiments showed that SGLT2 inhibitors may contribute to improvement of hepatic steatosis and liver function. Our results suggest that SGLT2 inhibitors also exert these effects in humans. Moreover, the effects on liver may be greater especially in patients with more serious liver dysfunction.

The present study has several limitations. First, other hypoglycemic, anti-hypertensive, or lipid lowering agents, food intakes and/or exercise levels may have an influence on the study results. Second, we did not consider the possible differences of each SGLT2 inhibitor. Although it has been thought that SGLT2 inhibitors have similar effects because of its similarity on the chemical structures, further investigations will be needed. Third, the number of studied subjects was small because of the limited availability. Fourth, since the study was based on charts, lack of data might influence the results. Finally, while proposing the possible improvement of liver function by SGLT2 inhibitors, imaging tests might have been useful for more precise evaluation. A more detailed prospective study is recommended to evaluate the effects of SGLT2 inhibitors on metabolic parameters more validly.

### Conclusion

We studied the effects of SGLT2 inhibitors on metabolic parameters in patients with type 2 diabetes, and found that SGLT2 inhibitors significantly reduced HbA1c and body weight, and also improved liver function. SGLT2 inhibitors are more effective to improve glycemic control and liver function in diabetic patients with higher HbA1c and ALT levels.
